# Fluorescence-guided laparoscopic lymph node biopsy in a lymphoma patient: a possible new clinical application of indocyanine green

**DOI:** 10.1093/jscr/rjac047

**Published:** 2022-03-09

**Authors:** Marco Casaccia, Tommaso Testa, Sofia Paola Martigli, Matteo Santoliquido, Roberto Massimo Lemoli

**Affiliations:** Surgical Clinic Unit I, Department of Surgical Sciences and Integrated Diagnostics (DISC), University of Genoa, Genoa, Italy; Surgical Clinic Unit I, Department of Surgery, San Martino Hospital, Genoa, Italy; Surgical Clinic Unit I, Department of Surgical Sciences and Integrated Diagnostics (DISC), University of Genoa, Genoa, Italy; Surgical Clinic Unit I, Department of Surgery, San Martino Hospital, Genoa, Italy; Department of Internal Medicine (DiMI), Clinic of Hematology, University of Genoa, Genoa, Italy

**Keywords:** indocyanine green, ICG, laparoscopic surgery, laparoscopic biopsy, lymphoma

## Abstract

To date, there are no reports indicating the use of indocyanine green (ICG) fluorescence to detect pathologic lymphatic tissue when a laparoscopic lymph node biopsy (LLB) for suspected new or recurrent lymphoma is performed. We present the case of a 72-year-old female patient admitted for suspicion of recurrent lymphoma. A preoperative imaging work-up showed solid tissue enveloping the terminal portion of the abdominal aorta with a standardized uptake value (SUV) of 10. Therefore, an LLB was planned. After induction of anesthesia, a ICG solution was injected intravenously and subcutaneously at both inguinal regions. At laparoscopy, a complete visualization of the pathologic lymph nodes was achieved, enabling an incisional biopsy of the lymphomatous mass. LLB with ICG-fluorescence offers a simple and safe method for pathologic lymph node detection in the suspicion of intra-abdominal lymphoma. More studies with large case series are needed to confirm the efficacy of this application.

## INTRODUCTION

The value of near-infrared (IR) indocyanine green (ICG) fluorescence-guided surgery is now established in the colo-rectal and gastric surgery as well as in breast surgery and in the gynecological field [1–3]. Real-time visualization of lymphatic mapping can help identify the pattern and status of lymph node basin implementing the concept of tailored lymphadenectomy. When new onset or recurrence of lymphoproliferative disease is suspected, a surgical biopsy of the pathological lymph nodes is required to establish the diagnosis [4–6].

When lymphadenopaties are deeply located in the abdomen, a needle biopsy is often not possible due to the proximity to large vessels and the risk of bleeding. In this setting, a laparoscopic biopsy is the correct option [7]. Until now, no records are reported in literature concerning the use of ICG for the diagnosis of lymphoproliferative diseases with involvement of the abdominal lymph node stations. In this case, we describe a laparoscopic biopsy of periaortic pathologic lymph nodes detected by intraoperative ICG fluorescence.

## CASE REPORT

**Figure 1:**
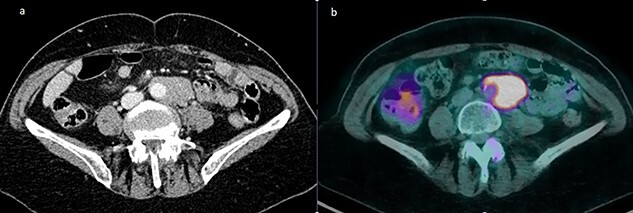
(**a**) Solid tissue enveloping the terminal portion of the abdominal aorta measuring approximately 45 × 26 × 81 mm in diameters; (**b**) fluorodeoxyglucose PET showing a pathological uptake with SUV of 10 at the level of the para-aortic lymphomatous tissue.

A 72-year-old female patient was hospitalized for suspicion of recurrent intra-abdominal lymphoma. Prior diagnosis was a B-cell follicular non-Hodgkin lymphoma diagnosed in 2017 and treated with chemotherapy with a good response. A positron emission tomography/computed tomography (PET/CT) scan showed solid tissue enveloping the terminal portion of the subrenal abdominal aorta and the left common iliac artery, measuring approximately 45 × 26 × 81 mm in diameters (Fig. 1a). A hyperaccumulation of the tracer with a standardized uptake value (SUV) of 10 was shown in correspondence with the known tissue of probable lymphomatous origin (Fig. 1b). Further uptake in the subcentimetric left common iliac lymphadenopaties was present as well. Therefore, a laparoscopic lymph node biopsy (LLB) was planned for diagnostic purposes in the suspicion of a relapse of the lymphoproliferative disease. 

After induction of anesthesia, ICG (Verdye, Diagnostic Green GmbH, Aschheim-Dornach, Germany) was administered to the patient placed in the lithotomy position. The concentration used was 2.5 mg/ml. A 25-mg vial with ICG powder was diluted in 10 cc of aqueous sterile water. Three milliliters of this ICG solution was injected subcutaneously at both inguinal regions. The pneumoperitoneum was achieved through the umbilicus via an open technique; a 10-mm trocar was inserted to accommodate a 30°-angle telescope. A 10-mm trocar was inserted in the right iliac fossa and a 5-mm trocar was inserted in the right hypocondrium. Para-aortic lymphadenopathy was reached by an incision of the peritoneum covering the superior part of the right common iliac artery. This incision was prolonged along the left aspect of the aorta just above the inferior mesenteric artery. A Thunderbeat™ (Olympus Medical Systems Corp, Tokyo, Japan) device was used for dissection.

**Figure 2:**
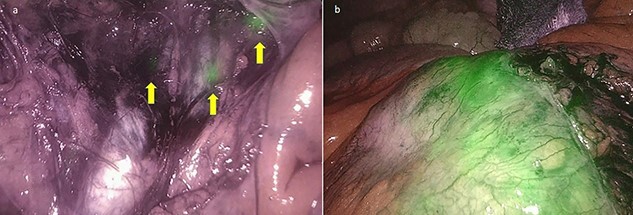
(**a**) Lymph nodes stained with ICG in the subserosa, surrounding the left iliac artery (yellow arrows); (**b**) the enhanced lymphomatous tumor after intravenous ICG administration at near-IR fluorescence vision.

**Figure 3:**
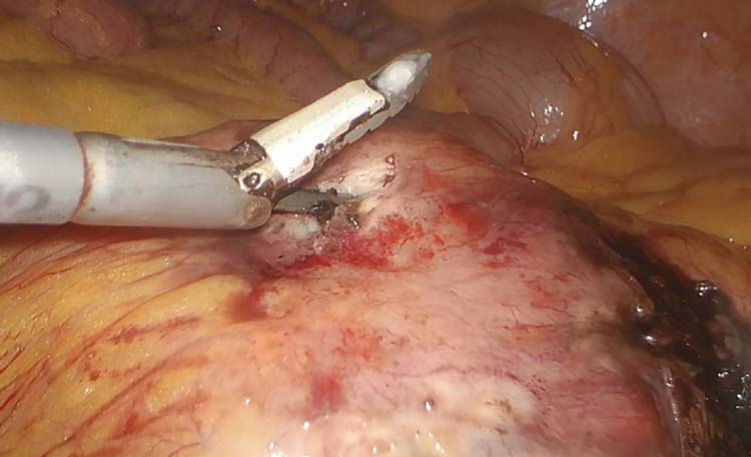
Incisional biopsy of the tumor realized by means of the Thunderbeat dissector.

A dedicated clinical endoscopic system (Visera Elite II, Olympus Medical Systems Corp, Tokyo, Japan) equipped with IR light source and IR UHD telescope was used to illuminate regional lymph nodes. Twenty minutes after the injection, lymph nodes stained with ICG were observed in the subserosa, surrounding the left iliac artery (Fig. 2a). Pathologic nodes came into contact with the known mass. Three milliliters of the above-mentioned solution were then administered intravenously, and after few minutes, the staining of the mass was evident (Fig. 2b). An incisional biopsy in two different areas of the tumoral mass was made (Fig. 3). The operation was completed laparoscopically. The post-operative course was uneventful and the patient was discharged at post-operative day1. The pathologic exam showed a localization of a peripheral B-cells non-Hodgkin lymphoma of centrofollicular origin compatible with follicular lymphoma.

## DISCUSSION

The use of intra-operative near-IR ICG fluorescence imaging has been recently described as a very useful tool in decision-making strategy during challenging surgical procedures. The real-time visualization of lymphatic flow (lymphatic mapping) has proven to be feasible, potentially aiding the surgeon to identify sentinel lymph nodes and to perform a better lymphadenectomy [8].

In the suspicion of lymphoproliferative disease and when isolated abdominal lymphadenopathy is poorly accessible to percutaneous biopsy, laparoscopy is the key tool for making diagnosis [4–6]. Para-aortic and pericaval lymph nodes are often affected by the onset of the disease, and when they are the only sites involved, a laparoscopic biopsy approach could be the right option [9].

Our report is the first to describe the use of ICG fluorescence in the search for pathological lymph nodes in the suspicion of lymphoproliferative disease originating from the abdominal lymph nodes. In this pathological condition, however, the issue of whether the dye is retained by the pathological lymph node as the healthy one is not known up to now. In colo-rectal or gastric cancer, after the peritumoral injection of ICG, the dye follows the lymphatic pathways and colors the lymph nodes it finds along the collectors. The presence of cancer cells within the metastatic lymph node does not seem to decrease the intensity of staining [10].

However, this statement is not univocal. Widespread lymph node metastases have been observed to induce an obstruction of lymphatic channels, and lymphatic drainage is bypassed to other (nonsentinel) lymph nodes [11]. In our case, the iliac lymph nodes visualized at PET as pathological, at laparoscopy, turned out to be stained up close to the large lymphomatous lesion. The latter, however, did not stain with ICG. Only after intravenous ICG administration, staining of the mass was evident. Histopathological examination showed that the tissue biopsied did not belong to a lymph node but it was extranodal tissue. This could explain the lack of vital staining after subcutaneous administration only. Since the lymphomatous mass had a marked metabolism, its visualization by intravenous ICG administration was possible; therefore, in this way, the combined administration of subcutaneous and intravenous ICG allowed to highlight both the pathological lymph nodes and the extra-nodal lymphomatous tissue.

Furthermore, the coupling of CT images to PET images is essential for surgical planning and execution. Accordingly, intraoperative staging, surgical planning and execution can be improved since determination of the completion and efficacy of surgical biopsy are targeted to vital, ICG-enhanced tissue.

In conclusion, ICG-enhanced fluorescence seems to provide several advantages in LLB, allowing both to better identify the surgical anatomy and to reduce the surgical time. The patients who could benefit most would be those in whom dissection could be dangerous due to proximity to the great vessels, or visualization of lymphadenopathies could be difficult because of their deep location. Our report, although encouraging, needs to be supported by more studies with large case series before considering this novel application of ICG-enhanced fluorescence in LLB as completely reliable.

## CONFLICT OF INTEREST STATEMENT

None declared.

## FUNDING

None.

## AUTHORS’ CONTRIBUTIONS

All authors have accepted responsibility for the entire content of this manuscript and have approved its submission.

## ETHICAL APPROVAL

The local Institutional Review Board deemed the study exempt from review.
